# The Dynamic Response and Vibration of Functionally Graded Carbon Nanotube-Reinforced Composite (FG-CNTRC) Truncated Conical Shells Resting on Elastic Foundations

**DOI:** 10.3390/ma10101194

**Published:** 2017-10-18

**Authors:** Duc Nguyen Dinh, Pham Dinh Nguyen

**Affiliations:** 1 Advanced Materials and Structures Laboratory, VNU-Hanoi, University of Engineering and Technology (UET-VNU), 144 Xuan Thuy, Cau Giay, Hanoi 100000, Vietnam; nguyenpd_58@vnu.edu.vn; 2 National Research Laboratory, Department of Civil and Environmental Engineering, Sejong University, 209 Neungdong-ro, Gwangjin-gu, Seoul 05006, Korea

**Keywords:** FG-CNTRC truncated conical shells, dynamic response and vibration, classical shell theory, elastic foundations

## Abstract

Based on the classical shell theory, the linear dynamic response of functionally graded carbon nanotube-reinforced composite (FG-CNTRC) truncated conical shells resting on elastic foundations subjected to dynamic loads is presented. The truncated conical shells are reinforced by single-walled carbon nanotubes (SWCNTs) that vary according to the linear functions of the shell thickness. The motion equations are solved by the Galerkin method and the fourth-order Runge–Kutta method. In numerical results, the influences of geometrical parameters, elastic foundations, natural frequency parameters, and nanotube volume fraction of FG-CNTRC truncated conical shells are investigated. The proposed results are validated by comparing them with those of other authors.

## 1. Introduction

In the past few decades, carbon nanotubes (CNTs) have generated huge research interest from many areas of science and engineering. This is mainly due to their remarkable structure: CNTs are allotropes of carbon with a cylindrical nanostructure [1,2]. These cylindrical carbon molecules have unusual properties, which are valuable for nanotechnology, electronics, optics and other fields of materials science and technology. Numerous studies have shown that CNTs have excellent mechanical, electrical, and thermal properties. For example, CNTs’ materials are the strongest and stiffest materials yet discovered in terms of tensile strength and elastic modulus. This strength results from the covalent sp2 bonds formed between the individual carbon atoms. For example, a multi-walled carbon nanotube has a tensile strength of 63 GPa [3], while an individual CNT shell has a strength of up to 100 GPa [4], which is in agreement with quantum/atomistic models. In addition, CNTs have a low density for a solid of 1.3 to 1.4 g/cm^3^; the specific strength of up to 48,000 kN·m·kg^−1^ is the best of known materials, compared to high-carbon steel’s 154 kN·m·kg^−1^ [5]. About the electrical properties, because of CNTs’ nanoscale cross section, electrons propagate only along the tube’s axis. CNTs are one-dimensional conductors, or, in other words, metallic or semiconducting along the tubular axis. The maximum electrical conductance of a single-walled CNT is 4 e^2^/h, twice the conductivity of a single ballistic quantum channel [6]. As for the thermal properties, as far as we know, all nanotubes are expected to be very good thermal conductors along the tube; in fact, the temperature stability of CNTs is estimated to be up to 2800 °C in vacuum and about 750 °C in air [7]. Moreover, CNTs have optical properties such as useful absorption, photoluminescence (fluorescence), and Raman spectroscopy. The superior properties of CNTs are well established and have immediate applications in areas related to most industries, including aerospace, electronics, medicine, defense, automotive, energy, construction, and even fashion. For example, Aliahmad et al. presented the poly (vinylidene fluoride-hexafluoropropylene) porous membrane electrolyte enhanced with lithium bis(trifluoromethane sulphone)imide and lithium aluminum titanium phosphate with an ionic conductivity of 2.1 × 10^−3^ S·cm^−1^ for paper-based battery applications [8] and the paper–based lithium–ion batteries using carbon nanotube coated wood microfibers [9]. Agarwal et al. studied the conductive paper from lignocellulose wood microfibers coated with nanocomposite of carbon nanotubes and conductive polymers [10].

As a new breed of composite materials and the components of structures in the advanced engineering, FGMs (functionally graded materials) are microscopically inhomogeneous composites usually made of a mixture of metal and ceramic in which the volume ratios of each composition vary smoothly and continuously from one surface to another according to the structure thickness to be suitable to the specific strength of materials. For FGMs, the mechanical properties vary smoothly and continuously in preferred directions, which enables FGMs to avoid interface problems and unexpected thermal stress concentrations. There are many areas of application for FGM. The concept is to make a composite material by varying the microstructure from one material to another material with a specific gradient. This enables the microstructure to have the best characteristics of both materials. If it is for thermal or corrosive resistance or malleability and toughness, both strengths of the material may be used to avoid corrosion, fatigue, fractures, and stress corrosion cracking. Due to the high heat resistance, FGMs are appropriate to use as structural components operating in ultrahigh-temperature environments and subjected to extremely high thermal gradients, such as in nuclear plants, aerospace structures, aircraft, and other engineering applications. This has prompted considerable research focusing on linear and nonlinear analyses of FGMs in recent years.

As can be seen, both CNTs and FGMs are breakthroughs in materials science. If the texture is made of a material that is a combination of CNT and FGM, then the texture would have been better. The unique features of FGMs and CNTs may be combined, for instance, through functionally graded distributions of CNTs in a FGM media. Functionally graded carbon nanotube-reinforced composites (FG-CNTRC) were first introduced by Shen [11] and have recently become more popular.

FG-CNTRC could be embedded in beams, panels, plates, and shells as structural elements. By considering the temperature dependence of material properties and the initial thermal stresses, Amin et al. studied the free vibration behavior of pre-twisted FG-CNTRC beams in a thermal environment [12], in which the governing equations were derived based on the higher-order shear deformation theory of beams and the free vibration eigenvalue equations are extracted using the Chebyshev–Ritz method. Wu and his colleague analyzed the imperfection sensitivity of the post-buckling behavior of FG-CNTRC beam subjected to axial compression based on the first order shear deformation beam theory with a von Kármán geometric nonlinearity in [13] and analyzed thermal post-buckling behavior of FG-CNTRC beam subjected to in-plane temperature variation in [14]. 

In a study on the FG-CNTRC panels using the Chebyshev–Ritz method, first-order shear deformation shell theory, and Donnell-type kinematic assumptions, Miraei and Kiani presented free vibration FG-CNTRC cylindrical panels [15]. To investigate the effect of the main design variables that influence the linear buckling behavior of FG-CNTRC unstiffened curved panels, in [16] the authors presented a buckling analysis of FG CNT-reinforced curved panels under axial compression and shear. The analysis of flexural strength and free vibration of carbon nanotube-reinforced composite cylindrical panels was carried out in [17] by Liew et al., with four types of distributions of uniaxially aligned reinforcements. 

About the FG-CNTRC plates, based on iso-geometric analysis (IGA) and generalized higher-order shear deformation theory (GHSDT), in [18] Phung et al. performed a size-dependent analysis of FG-CNTRC nano-plates with nonlocal governing equations approximated according to IGA based on GHSDT, which naturally satisfies the higher-order derivatives continuity requirement in the weak form of FG-CNTRC nanoplates. By using first-order shear deformation plate theory, the authors of [19] presented a geometrically nonlinear analysis of FG-CNTR composite laminated plates (which were composed of perfectly bonded carbon nanotube-reinforced functionally graded layers; in each layer, CNTs are assumed to be uniformly distributed or functionally graded in the thickness direction) by using first-order shear deformation plate theory and the Von Kármán assumption accounting for transverse shear strains, rotary inertia, and moderate rotations. Kiani analyzed the free vibration behavior of FG-CNTRC plates integrated with piezoelectric layers at the bottom and top surfaces [20], while the buckling behavior of FG CNTR composite laminated plate was studied in [21], in which the first-order shear deformation theory (FSDT) was employed to incorporate the effects of rotary inertia and transverse shear deformation, and the meshless kp-Ritz method was used to obtain the buckling solutions. By applying the traditional Galerkin method and the Airy stress function, Duc et al. [22] presented the static response and free vibration of functionally graded carbon nanotube-reinforced composite rectangular plates resting on Winkler–Pasternak elastic foundations; Thanh et al. [23] studied the nonlinear dynamic response and vibration of FG-CNTRC shear deformable plates with temperature-dependent material properties and surrounded by elastic foundations.

The FG-CNTRC shells are also of interest to researchers; however, the number of studies is still limited. For example, the free vibration characteristics of embedded FG-CNTRC spherical shells were studied [24] based on a numerical approach according to the first-order shear deformation shell theory and by using differential operators. Ansari and colleague presented a nonlinear post-buckling analysis of piezoelectric FG-CNTRC cylindrical shells subjected to combined electro-thermal loadings, axial compression and lateral loads in [25] by applying the Ritz energy approach. Based on the theory of elasticity, static analysis of FG-CNTRC cylindrical shell imbedded in piezoelectric sensor and actuator layers under thermo-electro-mechanical load was carried out by Alibeigloo and Pasha Zanoosi [26]. In particular, up to this point, studies on conical and truncated conical FG-CNTRC include: according to the adjacent equilibrium criterion, [27] dealt with the buckling of FG-CNTRC conical shells subjected to pressure loading using first-order theory of shells and geometrical non-linearity of von-Karman and Donnell kinematic assumptions; Duc et al. [28] studied the linear thermal and mechanical instability of the FG-CNTRC truncated conical shells reinforced by CNT fibers and surrounded by elastic foundations in a thermal environment, with the equilibrium and linearized stability equations for the shells derived based on the classical shell theory; Mirzaei and Kiani [29] studied the thermal buckling of FG-CNTRC conical shells with axially immovable edge supports subjected to uniform temperature rise loading in. Hamilton’s principle and the differential quadrature method (DQM) were employed to discretize the governing differential equations subjected to the related boundary conditions for studying free vibration analysis of rotating FG-CNTRC truncated conical shells in [30] by Heydarpour et al., and Reza and Jalal studied the buckling and vibration of FG-CNTRC conical shells under axial loading [31]. Via the extended Hamilton principle based on the basis of Novozhilov nonlinear shell theory and Green–Lagrange geometrical nonlinearity and using Fourier expansion and the HDQ discretization, Mehri et al. studied the buckling and vibration of the FG-CNTRC truncated conical shell simultaneously subjected to axial compression and external pressure in [32] and dealt with the dynamic instability of a pressurized functionally graded carbon nanotube-reinforced truncated conical shell subjected to yawed supersonic airflow in [33]. Additional investigations on FG-CNTRC structures are also reported in the literature [34,35,36,37,38,39,40,41,42,43].

Truncated conical shells are one of the principal elements of structure in many technical fields. For instance, they are used for aircraft, satellites, submarines, and water-borne ballistic missiles; in civil engineering, they are frequently used in containment vessels in elevated water tanks. In the open-source literature, there are several authors who have studied linear and nonlinear conical cones and truncated cones made of different materials; the best-known author is Sofiyev, some of whose investigations are reported in the literature [44,45,46,47,48,49,50,51,52,53]. 

Despite all the abovementioned studies, there has been no work on the structural responses of the analysis of FG-CNTRC truncated conical shells resting on Winkler and Pasternak’s elastic foundations using the analytic method. Thus, this study is indispensable for understanding the structural responses of FG-CNTRC truncated conical shells.

In the present work, by using the classical thin shell theory, an approximate solution, which was proposed by Agamirov [54] and used by Sofiyev [55] for FGM truncated conical shells and Duc et al. in [56] for FGM annular spherical shells, the authors tried to apply this form to solve problems related to FG-CNTRC truncated conical shells. The object of the present investigation is to give analytical solutions to the problem of the dynamic response of FG-CNTRC truncated conical shells resting on elastic foundations.

## 2. Formulation of the Problem

**Figure 1:**
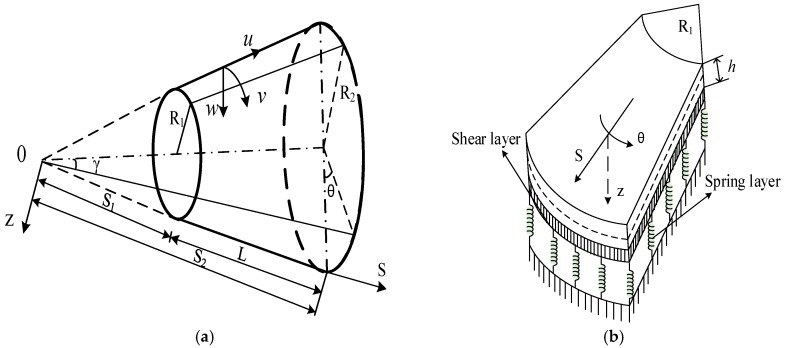
(**a**) The coordinate system and geometric characteristics of a FG-CNRTC truncated conical shell; (**b**) the geometric characteristics of the truncated conical shell surrounded by elastic foundations.

Consider a thin FG-CNTRC truncated conical shells surrounded by elastic foundations, with a thickness of shell h, and radii $R_{1}<R_{2}$, length L and the semi-vertex angle of the cone γ. The meridional, circumferential, and normal directions of the shell are denoted by S,θ and z, respectively. A schematic of the shell with the assigned coordinate system and geometric characteristics is shown in Figure 1.

### 2.1. Determination of the Elastic Modules of CNTRCs and FG-CNTRC

**Figure 2:**
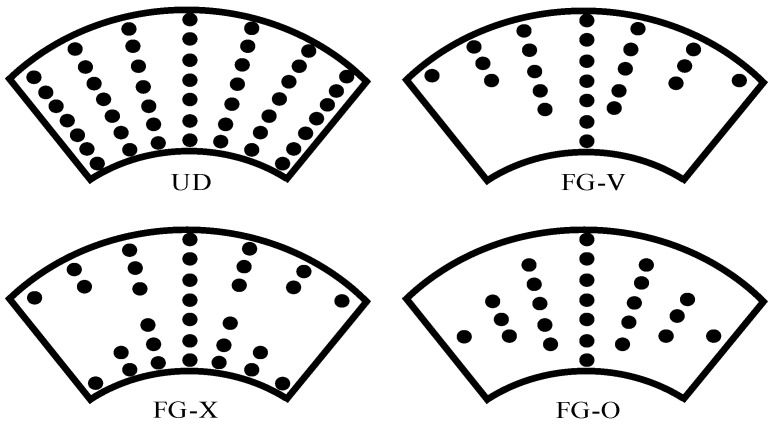
Configurations of various CNTRC truncated conical shells.

In the present study, the FG-CNTRC material is made of poly(methyl methacrylate), referred to as PMMA, reinforced by (10,10) single-walled carbon nanotubes (SWCNT). The SWCNT reinforcement is either uniformly distributed (UD) or functionally graded (FG) in the thickness direction [11,12]. FG-V, FG-X, and FG-O CNTRC (Figure 2) are the functionally graded distribution of CNTs through the thickness direction of the composite truncated conical shell.

The elastic modules of the FG-CNTRC material are determined as follows [11]:

(1) $$\begin{array}{l} E_{11}=\eta_{1}V_{CNT}E_{11}^{CNT}+V_{m}E_{m},\\ \frac{\eta_{2}}{E_{22}}=\frac{V_{CNT}}{E_{22}^{CNT}}+\frac{V_{m}}{E_{m}},\\ \frac{\eta_{3}}{G_{12}}=\frac{V_{CNT}}{G_{12}^{CNT}}+\frac{V_{m}}{G_{m}}, \end{array}.$$

In the above equations, $E_{11}^{CNT},E_{11}^{CNT},G_{12}^{CNT}$ are the Young’s and shear modulus of the CNT, respectively; $E_{m},G_{m}$ are mechanical properties of the matrix, $\eta_{i}(i=\bar{1,3})$ are the CNT efficiency parameters and $V_{CNT},V_{m}$ are the volume fractions of the CNT and the matrix, respectively. The volume fractions of the CNT and the matrix are assumed to change according to the linear functions of the shell thickness. Specifically, the volume fractions of the CNT are expressed as follows:

(2) $$V_{CNT}=\left\{ \begin{array}{ll} V_{CNT}^{*} & \left(UD\right)\\ V_{CNT}^{*}\left(1+2\frac{z}{h}\right) & \left(FG-V\right)\\ 2V_{CNT}^{*}\left(1-2\frac{\left|z\right|}{h}\right) & \left(FG-O\right)\\ 4V_{CNT}^{*}\frac{\left|z\right|}{h} & \left(FG-X\right) \end{array}\right.,\;V_{m}=1-V_{CNT}.$$

The effective Poisson’s ratio and mass destiny may be written as [11,28]:

(3) $$\begin{array}{l} v_{12}=V_{CNT}^{*}v_{12}^{CNT}+V_{m}v^{m},\\ \rho =V_{CNT}\rho_{CNT}+V_{m}\rho_{m}, \end{array}$$

where $\left(\nu_{12}^{CNT},\rho_{CNT}\right)$ and $\left(\nu_{m},\rho_{m}\right)$ are Poisson’s ratio and mass destiny of the CNT and the matrix, respectively.

The CNT efficiency parameters $\eta_{i}(i=\bar{1,3})$ used in Equation (1) are estimated by matching Young’s modulus $E_{11}$ and $E_{22}$ and the shear modulus $G_{12}$ of FG-CNTRC material obtained by the extended rule of mixture to molecular simulation results. For various volume fraction of CNTs, these parameters are [11,28,33]: $\eta_{1}=0.137,\eta_{2}=1.022,\eta_{3}=0.715$ for the case of $V_{CNT}^{*}=0.12\left(12\%\right)$; $\eta_{1}=0.142,\eta_{2}=1.626,\eta_{3}=1.138$ for the case of $V_{CNT}^{*}=0.17\left(17\%\right)$ and $\eta_{1}=0.141,\eta_{2}=1.585,\eta_{3}=1.109$ for the case of $V_{CNT}^{*}=0.28\left(28\%\right)$.

### 2.2. Analytical Modeling of Elastic Medium

The FG-CNTRC truncated conical shell is surrounded by an elastic medium. The reaction–deflection relation of Pasternak foundation is given by [54,55]:

(4) $$q_{e}(S,\varphi )=K_{w}w-K_{p}\Delta w,$$

where φ=θsin(γ), $\Delta w=\left(\frac{\partial^{2}w}{\partial S^{2}}+\frac{1}{S}\frac{\partial w}{\partial S}+\frac{1}{S^{2}}\frac{\partial^{2}w}{\partial \varphi^{2}}\right)$, w is the deflection of the shell, $K_{w}\left(N/m^{3}\right)$ is the Winkler foundation modulus and $K_{p}\left(\text{N}/\text{m}\right)$ is the shear layer foundation stiffness of the Pasternak model.

### 2.3. Basic Formulation of the FG-CNTRC Truncated Conical Shells Surrounded by Elastic Foundations

The classical shell theory is used to obtain the motion and compatibility equations of thin FG-CNTRC truncated conical shell in this study. 

The strains across the shell thickness at a distance z from the mid-plane are:

(5) $$\left\{\begin{array}{l} \varepsilon_{S}\\ \varepsilon_{\theta }\\ \gamma_{S\theta } \end{array}\right\}=\left\{\begin{array}{l} \varepsilon_{S}^{0}\\ \varepsilon_{\theta }^{0}\\ \gamma_{S\theta }^{0} \end{array}\right\}+z\left\{\begin{array}{l} k_{S}^{}\\ k_{\theta }^{}\\ 2k_{S\theta }^{} \end{array}\right\}.$$

The strains at the middle surface and the change of curvatures and twist are related to the displacement components u,v,w in the S,φ,z coordinate directions, respectively [54,55,57]:

(6) $$\begin{array}{l} \varepsilon_{S}^{0}=\frac{\partial u}{\partial S},\\ \varepsilon_{\theta }^{0}=\frac{1}{S}\frac{\partial v}{\partial \varphi }+\frac{u}{S}-\frac{w}{S}\cot\gamma ,\\ \gamma_{S\theta }^{0}=\frac{1}{S}\frac{\partial u}{\partial \varphi }-\frac{v}{S}+\frac{\partial v}{\partial S},\; \end{array}\begin{array}{l} k_{S}=-\frac{\partial^{2}w}{\partial S^{2}},\\ k_{\theta }=-\frac{1}{S^{2}}\frac{\partial^{2}w}{\partial \varphi^{2}}-\frac{1}{S}\frac{\partial w}{\partial S},\\ k_{S\theta }=-\frac{1}{S}\frac{\partial^{2}w}{\partial S\partial \varphi }+\frac{1}{S^{2}}\frac{\partial w}{\partial \varphi }, \end{array}$$

where $\varepsilon_{S}^{0}$ and $\varepsilon_{\theta }^{0}$ are the normal strains in the curvilinear coordinate directions *S* and θ on the reference surface respectively, $\gamma_{S\theta }^{0}$ is the shear strain at the middle surface of the shell, and $k_{S},k_{\theta },k_{S\theta }$ are the changes of curvatures and twist. 

The geometrical compatibility equation of the shells is written as [54,55]:

(7) $$\frac{\cot\gamma }{S}\frac{\partial^{2}w}{\partial S^{2}}-\frac{1}{S}\frac{\partial^{2}\gamma_{S\theta }^{0}}{\partial S\partial \varphi }-\frac{1}{S^{2}}\frac{\partial \gamma_{S\theta }^{0}}{\partial \varphi }+\frac{\partial^{2}\varepsilon_{\theta }^{0}}{\partial S^{2}}+\frac{1}{S^{2}}\frac{\partial^{2}\varepsilon_{S}^{0}}{\partial \varphi^{2}}+\frac{2}{S}\frac{\partial \varepsilon_{\theta }^{0}}{\partial S}-\frac{1}{S}\frac{\partial \varepsilon_{S}^{0}}{\partial S}=0.$$

The stress—strain relations of the shell within the classical shell theory are given as:

(8) $$\left[\begin{array}{c} \sigma_{S}\\ \sigma_{\theta }\\ \sigma_{S\theta } \end{array}\right]=\left[\begin{array}{ccc} Q_{11} & Q_{12} & 0\\ Q_{12} & Q_{22} & 0\\ 0 & 0 & Q_{66} \end{array}\right]\left[\begin{array}{c} \varepsilon_{S}\\ \varepsilon_{\theta }\\ \gamma_{S\theta } \end{array}\right],$$

where the quantities $Q_{ij},\left(ij=11,12,22,66\right)$ are functions of non-dimensional thickness coordinates and are expressed as:

$$Q_{11}=\frac{E_{11}}{1-\nu_{12}\nu_{21}},Q_{22}=\frac{E_{22}}{1-\nu_{12}\nu_{21}},Q_{12}=\frac{\nu_{21}E_{11}}{1-\nu_{12}\nu_{21}},Q_{66}=G_{12}.$$

The force and moment resultants of FG-CNTRC shells are given by:

(9) $$\left(N_{i},M_{i}\right)=\overset{h/2}{\underset{-h/2}{\int }}\sigma_{i}(1,z)dz,\left(i=s,\theta \right).$$

Integrating the above stress–strain equations, the force and moment resultants of the shell are expressed in terms of the stress components through the thickness as:

(10) $$\left\{\begin{array}{c} N_{S}\\ N_{\theta }\\ N_{S\theta }\\ M_{S}\\ M_{\theta }\\ M_{S\theta } \end{array}\right\}=\left[\begin{array}{cccccc} A_{11} & A_{12} & 0 & B_{11} & B_{12} & 0\\ A_{12} & A_{22} & 0 & B_{12} & B_{22} & 0\\ 0 & 0 & A_{66} & 0 & 0 & 2B_{66}\\ B_{11} & B_{12} & 0 & D_{11} & D_{12} & 0\\ B_{12} & B_{22} & 0 & D_{12} & D_{22} & 0\\ 0 & 0 & B_{66} & 0 & 0 & 2D_{66} \end{array}\right]\left\{\begin{array}{c} \varepsilon_{S}^{0}\\ \varepsilon_{\theta }^{0}\\ \gamma_{S\theta }^{0}\\ k_{s}\\ k_{\theta }\\ k_{S\theta } \end{array}\right\},$$

where the coefficients $A_{ij},B_{ij},D_{ij}\left(i=1÷2,6;j=1÷2,6\right)$ are calculated by:

(11) $$\left(A_{ij},B_{ij},D_{ij}\right)=\overset{h/2}{\underset{-h/2}{\int }}Q_{ij}(1,z,z^{2})dz,\left(ij=11,12,22,66\right).$$

The motion equations of a truncated conical shell are based on the classical shell theory [54,55]:

(12) $$\begin{array}{l} S\frac{\partial N_{S}^{}}{\partial S}+\frac{\partial N_{S\theta }^{}}{\partial \varphi }+N_{S}^{}-N_{\theta }^{}=0,\\ \frac{\partial N_{\theta }^{}}{\partial \varphi }+S\frac{\partial N_{S\theta }^{}}{\partial S}+2N_{S\theta }^{}=0,\\ \frac{\partial^{2}M_{S}^{}}{\partial S^{2}}+\frac{2}{S}\frac{\partial M_{S}^{}}{\partial S}+\frac{2}{S}\left(\frac{\partial^{2}M_{S\theta }^{}}{\partial S\partial \varphi }+\frac{1}{S}\frac{\partial M_{S\theta }^{}}{\partial \varphi }\right)+\frac{1}{S^{2}}\frac{\partial^{2}M_{\theta }^{}}{\partial \varphi^{2}}-\frac{1}{S}\frac{\partial M_{\theta }^{}}{\partial S}-\frac{N_{\theta }^{}}{S}\cot\gamma \\ +q-K_{w}w+K_{p}\Delta w=I_{0}\frac{\partial^{2}w}{\partial t^{2}}, \end{array}$$

where $I_{0}$ is a parameter of density and is given by:

(13) $$I_{0}=\int_{-\frac{h}{2}}^{\frac{h}{2}}\rho dz.$$

The first two equations of the system of Equation (12) are identically satisfied by introducing an Airy stress function F(s,θ,t) as follows [54,55,57]:

(14) $$N_{S}=\frac{1}{S^{2}}\frac{\partial^{2}F}{\partial \varphi^{2}}+\frac{1}{S}\frac{\partial F}{\partial S},N_{\theta }=\frac{\partial^{2}F}{\partial S^{2}},N_{S\theta }=-\frac{1}{S}\frac{\partial^{2}F}{\partial S\partial \varphi }+\frac{1}{S^{2}}\frac{\partial F}{\partial \varphi }.$$

The reverse relations are obtained from Equation (10); one can write:

(15) $$\begin{array}{l} \varepsilon_{S}^{0}=A_{22}^{*}\left(\frac{1}{S^{2}}\frac{\partial^{2}F}{\partial \varphi^{2}}+\frac{1}{S}\frac{\partial F}{\partial S}\right)-A_{12}^{*}\left(\frac{\partial^{2}F}{\partial S^{2}}\right)+C_{11}^{}\frac{\partial^{2}w}{\partial S^{2}}+C_{21}^{*}\left(\frac{1}{S^{2}}\frac{\partial^{2}w}{\partial \varphi^{2}}+\frac{1}{S}\frac{\partial w}{\partial S}\right),\\ \varepsilon_{{}^{\theta }}^{0}=A_{11}^{*}\left(\frac{\partial^{2}F}{\partial S^{2}}\right)-A_{12}^{*}\left(\frac{1}{S^{2}}\frac{\partial^{2}F}{\partial \varphi^{2}}+\frac{1}{S}\frac{\partial F}{\partial S}\right)+C_{12}^{}\frac{\partial^{2}w}{\partial S^{2}}+C_{22}^{}\left(\frac{1}{S^{2}}\frac{\partial^{2}w}{\partial \varphi^{2}}+\frac{1}{S}\frac{\partial w}{\partial S}\right),\\ \gamma_{S\theta }^{0}=-A_{66}^{*}\left(\frac{1}{S}\frac{\partial^{2}F}{\partial S\partial \varphi }-\frac{1}{s^{2}}\frac{\partial F}{\partial \varphi }\right)+2C_{31}\left(\frac{1}{S}\frac{\partial^{2}w}{\partial S\partial \varphi }-\frac{1}{S^{2}}\frac{\partial w}{\partial \varphi }\right), \end{array}$$

where:

$$\begin{array}{l} A_{11}^{*}=\frac{A_{11}}{\Delta },A_{22}^{*}=\frac{A_{22}}{\Delta },A_{12}^{*}=\frac{A_{12}}{\Delta },A_{66}^{*}=\frac{1}{A_{66}},\Delta =A_{11}A_{22}-A_{12}^{2},\\ C_{11}=B_{11}A_{22}^{*}-B_{12}A_{12}^{*},C_{12}=B_{12}A_{11}^{*}-B_{11}A_{12}^{*},C_{13}=\left(B_{11}B_{11}^{*}+B_{12}B_{21}^{*}-D_{11}\right),\\ C_{14}=\left(B_{11}B_{12}^{*}+B_{12}B_{22}^{*}-D_{12}\right),C_{21}=B_{12}A_{22}^{*}-B_{22}A_{12}^{*},C_{22}=B_{22}A_{11}^{*}-B_{12}A_{12}^{*},\\ C_{23}=\left(B_{12}B_{11}^{*}+B_{22}B_{21}^{*}-D_{11}\right),C_{24}=\left(B_{12}B_{12}^{*}+B_{22}B_{22}^{*}-D_{12}\right),\\ C_{31}=B_{66}A_{66}^{*},C_{32}=2\left(B_{66}B_{66}^{*}-D_{66}\right). \end{array}$$

Substituting Equation (15) into Equations (7) and (10), and the third equation of the system of Equation (12), resulted in two new equations for *F* and *w.* For the simplicity of the mathematical operations, the variable $S=S_{1}e^{x}$ is included and $F=F_{1}e^{2x}$ is taken into account instead of F. After lengthy computations, the system of partial differential equations for $F_{1}$ and w can be written in the form

(16) $$\left[\begin{array}{cc} L_{11} & L_{12}\\ L_{21} & L_{22} \end{array}\right]\left[\begin{array}{c} F_{1}\\ w \end{array}\right]=\left[\begin{array}{c} 0\\ 0 \end{array}\right],$$

with $L_{ij}$ given in Appendix A.

Equation (16) is the basic equation used to investigate the dynamic response of FG-CNTRC truncated conical shells. It is in terms of two dependent unknowns, w and $F_{1}$.

### 2.4. The Solution of Basic Equations

In this section, an analytical approach is used to investigate the dynamic response of shells resting on an elastic foundation. The shell is assumed to be simply supported at both edges of the shell.

(17) $$w=0\;\mathrm{at}\;x=0\;\mathrm{and}\;x=x_{0}.$$

The boundary conditions can be satisfied when the deflection w is approximated as follows [54,55,58]:

(18) $$w\left(x,\theta ,t\right)=f\left(t\right)e^{x}\sin\left(m_{1}x\right)\sin\left(m_{2}\varphi \right),$$

in which $m_{1}=\frac{m\pi }{x_{0}},m_{2}=\frac{n}{\sin\gamma },x_{0}=\ln\left(\frac{S_{2}}{S_{1}}\right)$, and f(t) is time dependent unknown function of the deflection, m is the number of half waves along a generatrix, and n is the number of full waves along a parallel circle.

By introducing Equation (18) into Equation (16) and solving the obtained equation by applying the superposition principle, the stress function can be obtained as

(19) $$F_{1}=f(t)\left[\begin{array}{l} K_{1}\sin\left(m_{1}x\right)+K_{2}\cos\left(m_{1}x\right)\\ +K_{3}e^{-x}\sin\left(m_{1}x\right)+K_{4}e^{-x}\cos\left(m_{1}x\right) \end{array}\right]\sin\left(m_{2}\varphi \right),$$

where the following definitions apply:

$$\begin{array}{l} K_{1}=\frac{m_{1}S_{1}\left(a_{1}m_{1}+a_{2}\right)\cot\gamma }{a_{2}^{2}+a_{1}^{2}},K_{2}=-\frac{m_{1}S_{1}\left(a_{1}-a_{2}m_{1}\right)\cot\gamma }{a_{2}^{2}+a_{1}^{2}},\\ K_{3}=-\frac{a_{3}a_{5}-a_{4}a_{6}}{a_{3}^{2}+a_{4}^{2}},K_{4}=-\frac{a_{3}a_{6}+a_{4}a_{5}}{a_{3}^{2}+a_{4}^{2}}, \end{array}$$

in which the remaining constants $a_{i}\left(i=1÷7\right)$ are given in Appendix B.

Applying the Galerkin method with the limits of integral is given by the formula:

(20) $$\overset{x_{0}}{\underset{0}{\int }}\overset{2\pi \sin\gamma }{\underset{0}{\int }}\left[L_{11}F_{1}+L_{12}w\right]e^{x}\sin(m_{1}x)\sin(m_{2}\varphi )d\varphi dx=0.$$

After substituting Equations (18) and (19) into Equation (16) and applying the Galerkin method as shown in Equation (20), we obtain the following equation:

(21) $$M_{5}I_{0}\frac{d^{2}f\left(t\right)}{dt^{2}}+\left(M_{1}+M_{2}K_{w}+M_{3}K_{p}+M_{4}\right)f\left(t\right)=q,$$

where the constants $M_{i}\left(i=1÷5\right)$ are given in Appendix C.

### 2.5. Vibration Analysis 

Assume that a FG-CNTRC truncated conical shell is acted on by a uniformly external pressure load q=QsinΩt.

Equation (21) is used to determine the dynamic response and vibration of FG-CNTRCs truncated conical shells under uniform external pressure. The dynamic response of the truncated conical shells can be gained by solving Equation (21), combined with the initial conditions $f\left(0\right)=0,\frac{df\left(0\right)}{dt}=0$, and using the Runge–Kutta method.

The linear free vibration for FG-CNRTC truncated conical shells without load form Equation (21) is obtained

(22) $$\frac{d^{2}f\left(t\right)}{dt^{2}}+\frac{M_{1}+M_{2}K_{w}+M_{3}K_{p}+M_{4}}{M_{5}I_{0}}f\left(t\right)=0.$$

The fundamental frequency of natural vibration of FG-CNRTC truncated conical shells can be expressed by:

(23) $$\omega_{F}=\sqrt{\frac{M_{1}+M_{2}K_{w}+M_{3}K_{p}+M_{4}}{M_{5}I_{0}}}.$$

The non-dimensional frequency of FG-CNRTC truncated conical shells is:

(24) $$\omega_{NF}=R_{1}\omega_{NF}\sqrt{\frac{\left(1-v_{m}^{2}\right)\rho_{m}}{E_{m}}}.$$

## 3. Numerical Results and Discussion

### 3.1. Validation

In order to validate the reliability of the method used in the paper, a comparison of the non-dimensional frequency is made with the results of other studies [59,60].

Table 1 shows the comparison of the non-dimensional frequency of the isotropic truncated conical shells with the same geometrical parameters $\Omega =\omega \sqrt{\frac{\rho \left(1-\nu^{2}\right)}{E}},\frac{R_{2}}{h}=100,\frac{L\sin\gamma }{R_{2}}=0.25,\nu =0.3,h=4mm,E=70GPa,\rho =2710\frac{Kg}{m^{3}}.$

In this table, the present results are compared with those of Li et al. [59] showing the calculations of natural frequencies and the forced vibration responses of conical shells using the Rayleigh–Ritz method. Lam and Hua presented the influence of boundary conditions on the frequency characteristics of a rotating truncated circular conical shell [60]. From this table, as can be seen, a good agreement is obtained in this comparison. A similar comparison is shown in [33].

### 3.2. The Natural Frequency and Dynamic Response 

The effects of CNT volume fraction, variously distributed types, and small radius to thickness ratio ($R_{1}/h$) on the non-dimensional frequency of the FG-CNTRC truncated conical shells are shown in Table 2 with the geometrical parameters $L/R_{1}=2,\gamma =30^{{}^{\circ}}.$ As can be seen, the value of the non-dimensional frequency increases when the value of $V_{CNT}^{*}$ increases and the non-dimensional frequency decreases when the value ratio $R_{1}/h$ increases. This is the case because when ratio $R_{1}/h$ increases, the truncated conical shell becomes thinner. In the case of variously distributed types of the CNTRC truncated conical shell, the non-dimensional frequency of the FG-X and FG-O types of shell are the highest and the lowest, respectively.

Table 3 shows the influence of semi-vertex angle γ and various types of CNTRC (FG and uniform) on the non-dimensional frequency of the truncated conical shells. It is clear that the value of the non-dimensional frequency decreases when the values of semi-vertex angle γ increase. The value of the non-dimensional frequency of the uniform distribution (UD) of CNTRC is consistently higher than one of the FG-CNTRCs (FG-O and FG-V). However, the value of the non-dimensional frequency of FG-X type is still highest, as shown in Table 2. 

**Figure 3:**
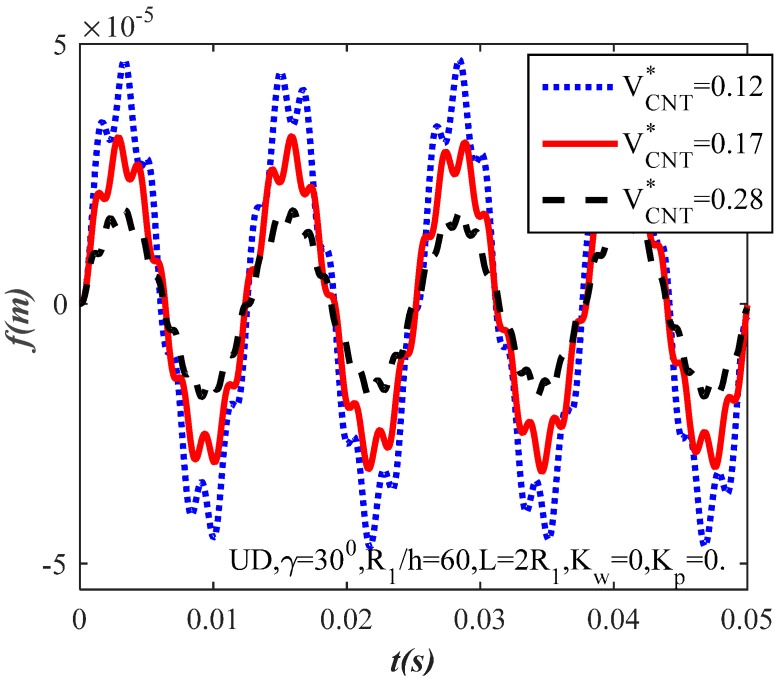
Effect of CNT volume fraction of fibers on the dynamic response of the CNTRC truncated conical shells with uniform distribution type.

**Figure 4:**
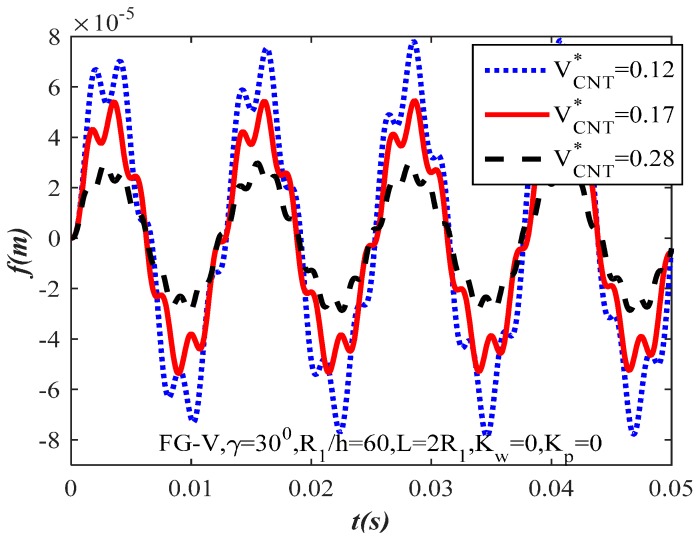
Effect of volume fraction of fibers on the dynamic response of the FG-CNTRC truncated conical shells.

Figure 3 and Figure 4 illustrate the influence of CNT volume fraction of fibers on the dynamic response of the CNTRC truncated conical shells. Three sets of CNT volume fraction are considered $V_{CNT}^{*}=\left(12\%,17\%,28\%\right)$. From these figures, as can be observed, the value of the shells’ amplitude increases when the CNT volume fraction decreases and vice versa. The CNT volume fraction increase makes the FG-CNTRC truncated conical shells have a better load capacity because the elastic modulus of the CNT is significantly stronger than the elastic modulus of the matrix. With the same geometrical parameters and the value of time, the amplitude of the uniform distribution CNTRC truncated conical shell (Figure 3) is considerably lower than the amplitude of the FG-V CNTRC truncated conical shell (Figure 4).

**Figure 5:**
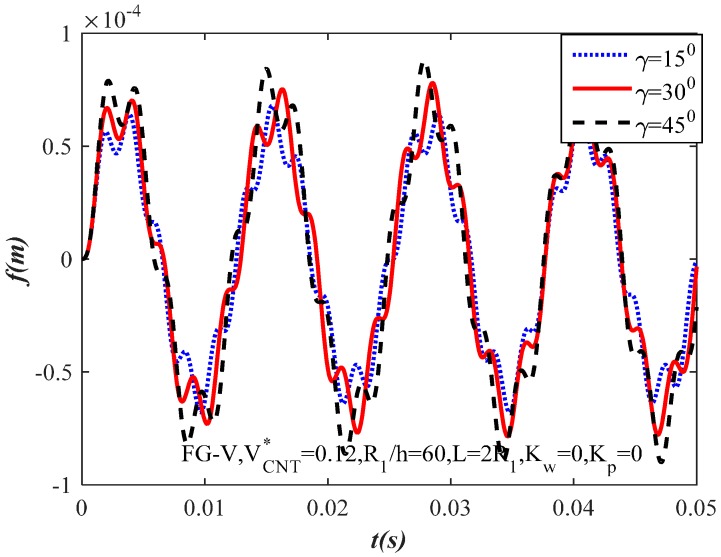
Effect of semi-vertex angle γ on the dynamic response of the FG-CNTRC truncated conical shells.

Figure 5 presents the influence of semi-vertex angle γ on the dynamic response of the FG-CNTRC truncated conical shells. Three different semi-vertex angles are considered. Increasing the value of the semi-vertex angles makes the value of the amplitude of the FG-CNTRC truncated conical shells increase. 

**Figure 6:**
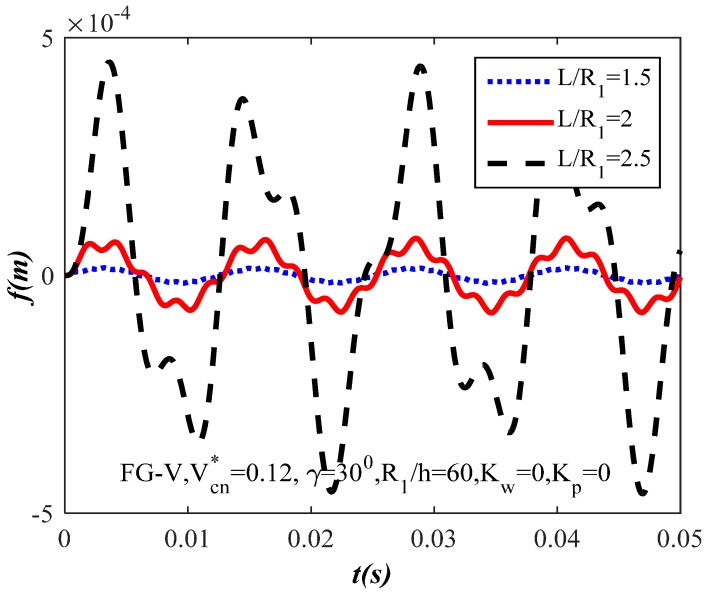
Effect of ratio $L/R_{1}$ on the dynamic response of the FG-CNTRC truncated conical shells.

Figure 6 shows the influence of ratio $L/R_{1}=\left(1.5,2,2.5\right)$ on the dynamic response of the FG-CNTRC truncated conical shells. From Figure 6, it is noticeable that when $L/R_{1}$ increases, the value of the shells’ amplitude increases and vice versa. 

**Figure 7:**
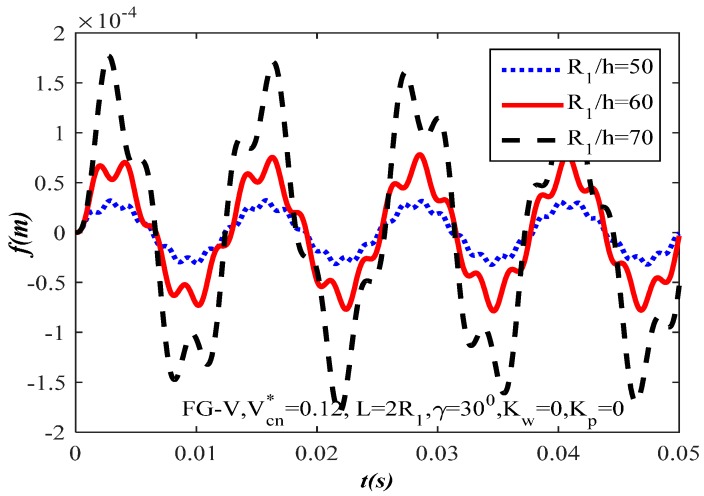
Effect of ratio $R_{1}/h$ on the dynamic response of the FG-CNTRC truncated conical shells.

The effect of ratio $R_{1}/h=\left(50,60,70\right)$ on the dynamic response of the FG-CNTRC truncated conical shells is shown in Figure 7. Clearly, the higher the ratio $R_{1}/h$, the higher the amplitude of the truncated conical shells. It is also understood that $R_{1}/h$ increase makes the FG-CNTRC truncated conical shells thinner which results in the lower the load capacity of the FG-CNTRC truncated conical shells.

**Figure 8:**
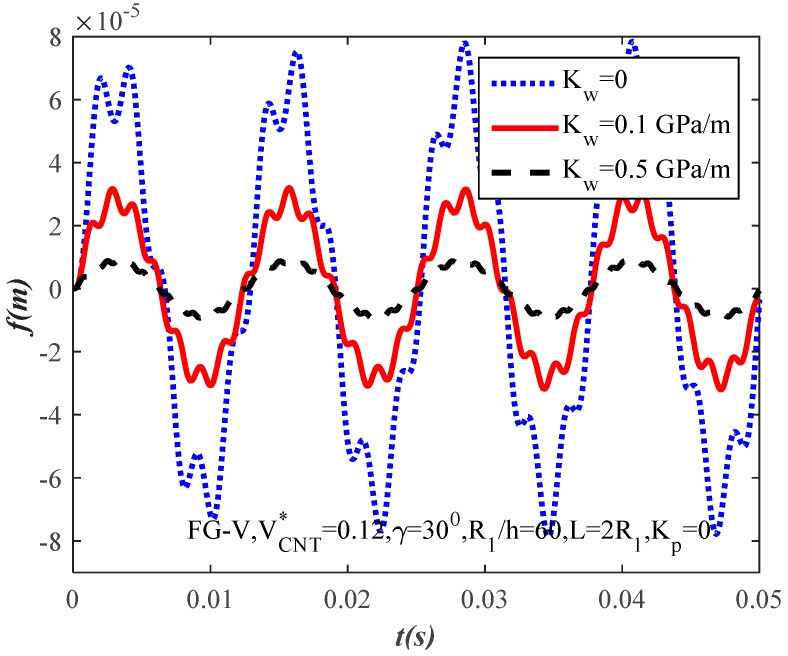
Effect of the Winkler modulus parameter $K_{w}$ on the dynamic response of the FG-CNTRC truncated conical shells.

**Figure 9:**
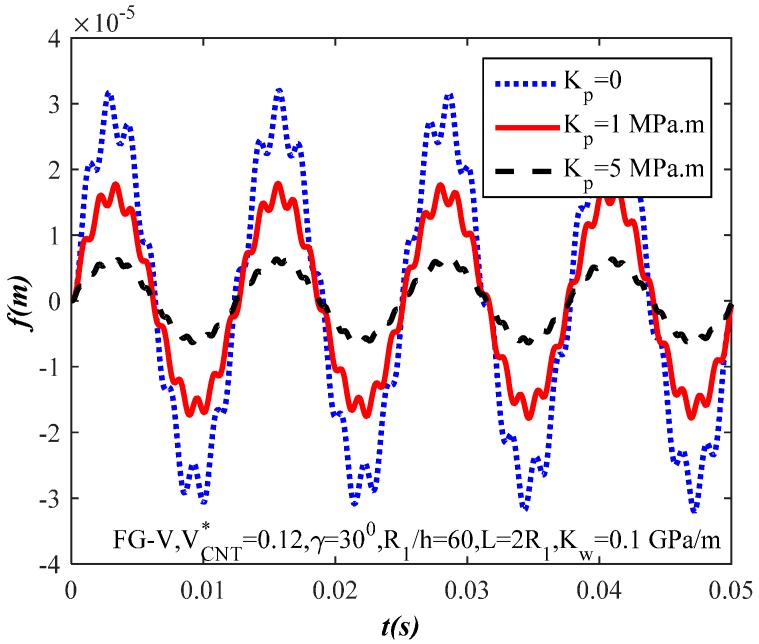
Effect of the Pasternak modulus parameter $K_{p}$ on the dynamic response of the FG-CNTRC truncated conical shells.

Figure 8 and Figure 9 show the effects of modulus $K_{w},K_{p}$ of the linear Winkler and Pasternak foundations, respectively. It is clear from the figures that the amplitude of the FG-CNTRC truncated conical shells decreases when the modulus of the elastic foundations increases. In other words, the elastic foundations have a positive effect on the reduction of the truncated conical shells’ amplitude. In conclusion, the load capacity is better when the FG-CNTRC truncated conical shells resting on elastic foundations.

**Figure 10:**
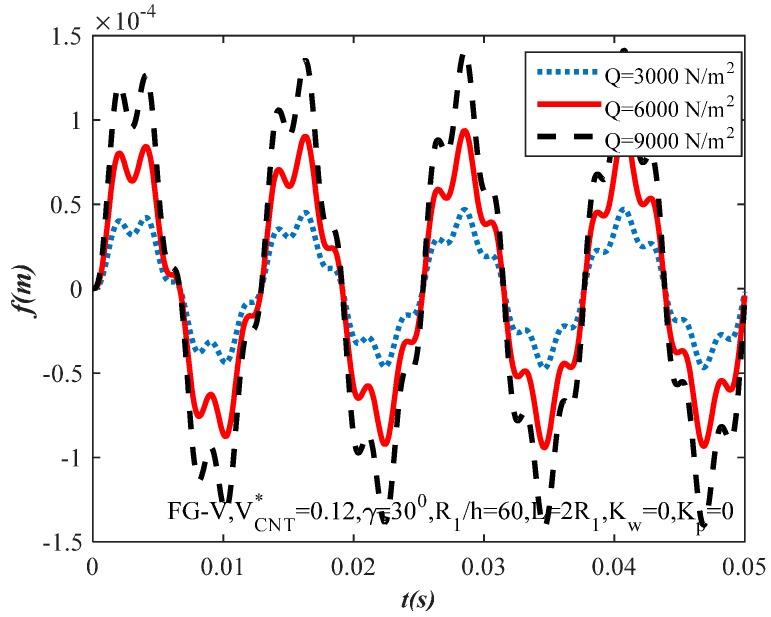
Effect of amplitude Q on the dynamic response of the FG-CNTRC truncated conical shells.

Figure 10 presents the effect of excitation force amplitude Q=(3000,6000,9000) on the dynamic response of the FG-CNTRC shells. It can be seen that an increase in the excitation force amplitude results in the increase of the FG-CNTRC truncated conical shells’ amplitude.

**Figure 11:**
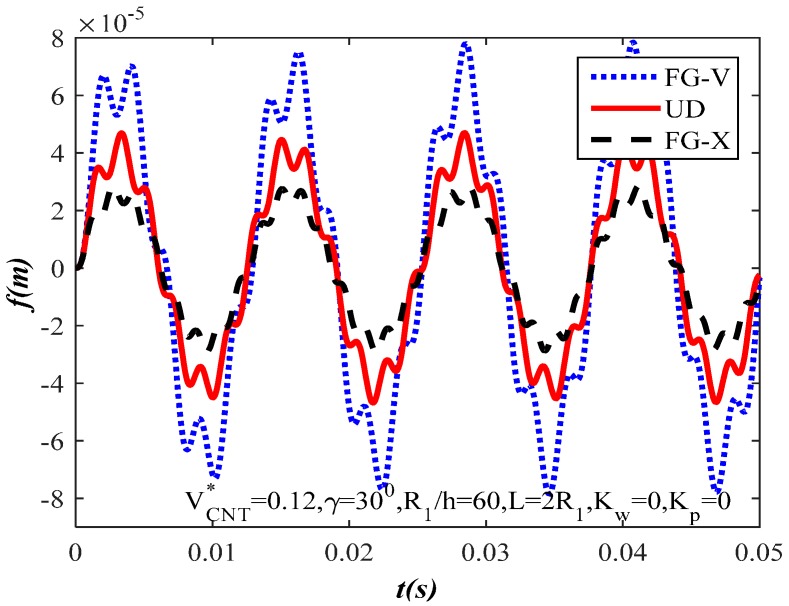
The dynamic response of CNTRC truncated conical shells with various types of CNT reinforcements.

Figure 11 illustrates the dynamic response of CNTRC truncated conical shells with three types of CNT reinforcements (FG-V, UD, and FG-X). It is noticeable that the various types of CNT distribution contribute to dramatic changes in amplitude. As we expected, the value amplitude of the FG-X type of CNT distribution is the smallest so the load capacity of the FG-X CNTRC truncated conical shells is the highest.

## 4. Conclusions

This paper studies the dynamic response and vibration of FG-CNTRC truncated conical shells resting on elastic foundations based on the classical shell theory. The following conclusions are obtained from this study:

- The value of the non-dimensional frequency negligible decreases when the values of semi-vertex angle γ increase.
- The value of the amplitude and non-dimensional frequency of the shells are significantly affected by various types of CNT distributions. In the case of the FG-X type of CNT distribution, the amplitude value is the smallest and the non-dimensional frequency is the highest.
- The results obtained also demonstrate that the t−f time–deflection curves are affected greatly by variations in parameters such as ratio $R_{1}/h$, length-to-radius ratio $L/R_{1}$, and amplitude Q.
- The elastic foundations strongly affect the dynamic response of FG-CNTRC truncated conical shells. The elastic foundations have positive effects on the amplitudes of FG-CNTRC truncated conical shells.
- The stress function, Galerkin method, Runge–Kutta method, and analytical approach are used to assess the dynamic responses of FG-CNTRC truncated conical shells resting on elastic foundations.

## Figures and Tables

**Table 1:** Comparisons of non-dimensional frequency parameter of the isotropic truncated conical shells.

γ	n	2	3	4	5	6
$30^{{}^{\circ}}$	Li et al. [59]	0.8431	0.7416	0.6419	0.5590	0.5008
	Lam and Hua [60]	0.8429	0.7376	0.6362	0.5528	0.4950
	Present	0.8700	0.7934	0.6831	0.5491	0.3976
$45^{{}^{\circ}}$	Li et al. [59]	0.7642	0.7211	0.6747	0.6336	0.6049
	Lam and Hua [60]	0.7655	0.7212	0.6739	0.6323	0.6035
	Present	0.7205	0.7023	0.6689	0.6149	0.5350

**Table 2:** Influences of CNT volume fraction, various types of FG-CNTRC and ratio $R_{1}/h$ on the non-dimensional frequency of the FG-CNTRC truncated conical shells.

$R_{1}/h$	$V_{CNT}^{*}=0.12$			$V_{CNT}^{*}=0.17$			$V_{CNT}^{*}=0.28$		
	FG-O	FG-V	FG-X	FG-O	FG-V	FG-X	FG-O	FG-V	FG-X
50	1.039	1.236	1.905	1.214	1.455	2.275	1.586	1.878	2.905
60	0.816	0.989	1.561	0.940	1.153	1.859	1.260	1.513	2.387
70	0.646	0.804	1.310	0.726	0.925	1.555	1.013	1.242	2.012
80	0.505	0.656	1.118	0.544	0.740	1.320	0.814	1.029	1.725
100	0.255	0.421	0.838	0.154	0.430	0.974	0.485	0.699	1.310

**Table 3:** Influences of semi-vertex angle γ on the non-dimensional frequency of the CNTRC truncated conical shells.

γ	UD	FG-O	FG-V	FG-X
15°	1.3672	0.8341	1.0526	1.7414
30°	1.2463	0.8160	0.9889	1.5606
45°	1.1507	0.7866	0.9311	1.4238
60°	1.0898	0.7658	0.8935	1.3371
75°	1.0567	0.7544	0.8731	1.2900

## Appendix A

$$\begin{array}{l} L_{11}\left(F_{1}\right)=C_{12}e^{2x}\frac{\partial^{4}F_{1}}{\partial x^{4}}+\left(C_{11}+4C_{12}-C_{22}\right)e^{2x}\frac{\partial^{3}F_{1}}{\partial x^{3}}\\ +\left(5C_{12}+3\left(C_{11}-C_{22}\right)-C_{21}\right)e^{2x}\frac{\partial^{2}F_{1}}{\partial x^{2}}+\left(2C_{12}+2\left(C_{11}-C_{22}\right)-2C_{21}\right)e^{2x}\frac{\partial F_{1}}{\partial x}\\ +\left(C_{11}+C_{22}-2C_{31}\right)e^{2x}\frac{\partial^{4}F_{1}}{\partial x^{2}\partial \varphi^{2}}+\left(C_{11}+3C_{22}-4C_{31}\right)e^{2x}\frac{\partial^{3}F_{1}}{\partial x\partial \varphi^{2}}\\ +\left(2C_{22}-2C_{31}+2C_{21}\right)e^{2x}\frac{\partial^{2}F_{1}}{\partial \varphi^{2}}+C_{21}e^{2x}\frac{\partial^{4}F_{1}}{\partial \varphi^{4}}-s_{1}^{}e^{3x}\left(\frac{\partial^{2}F_{1}}{\partial x^{2}}+3\frac{\partial F_{1}}{\partial x}+2F_{1}\right)\cot\gamma , \end{array}$$

$$\begin{array}{l} L_{12}\left(w\right)=C_{13}\frac{\partial^{4}w}{\partial x^{4}}+\left(-4C_{13}-C_{23}+C_{14}\right)\frac{\partial^{3}w}{\partial x^{3}}+\left(5C_{13}+3\left(C_{23}-C_{14}\right)-C_{24}\right)\frac{\partial^{2}w}{\partial x^{2}}\\ +\left(-2C_{13}+2\left(C_{14}-C_{23}\right)+2C_{24}\right)\frac{\partial w}{\partial x}+\left(-3C_{14}-\left(4C_{32}+C_{23}\right)\right)\frac{\partial^{3}w}{\partial x\partial \varphi^{2}}\\ +\left(C_{14}+2C_{32}+C_{23}\right)\frac{\partial^{4}w}{\partial x^{2}\partial \varphi^{2}}+\left(2C_{14}-2C_{32}+2C_{24}\right)\left(\frac{\partial^{2}w}{\partial \varphi^{2}}\right)\\ +C_{24}\left(\frac{\partial^{4}w}{\partial \varphi^{4}}\right)+s_{1}^{4}e^{4x}q-s_{1}^{4}e^{4x}K_{w}w+s_{1}^{2}e^{2x}K_{p}\left(\frac{\partial^{2}w}{\partial x^{2}}+\frac{\partial^{2}w}{\partial \varphi^{2}}\right)-I_{0}s_{1}^{4}e^{4x}\frac{\partial^{2}w}{\partial t^{2}}, \end{array}$$

$$\begin{array}{l} L_{21}\left(F_{1}\right)=A_{11}^{*}e^{2x}\frac{\partial^{4}F_{1}}{\partial x^{4}}+4A_{11}^{*}e^{2x}\frac{\partial^{3}F_{1}}{\partial x^{3}}+\left(5A_{11}^{*}-A_{22}^{*}\right)e^{2x}\frac{\partial^{2}F_{1}}{\partial x^{2}}\\ +\left(2A_{11}^{*}-2A_{22}^{*}\right)e^{2x}\frac{\partial F_{1}}{\partial x}+\left(2A_{66}^{*}-4A_{12}^{*}\right)e^{2x}\frac{\partial^{3}F_{1}}{\partial x\partial \varphi^{2}}\\ +\left(2A_{22}^{*}-2A_{12}^{*}+A_{66}^{*}\right)e^{2x}\frac{\partial^{2}F_{1}}{\partial \varphi^{2}}+A_{22}^{*}e^{2x}\frac{\partial^{4}F_{1}}{\partial \varphi^{4}}, \end{array}$$

$$\begin{array}{l} L_{22}\left(w\right)=C_{12}\frac{\partial^{4}w}{\partial x^{4}}+\left(C_{22}-C_{11}-4C_{12}\right)\frac{\partial^{3}w}{\partial x^{3}}+\left(5C_{12}-3C_{22}+3C_{11}-C_{21}\right)\frac{\partial^{2}w}{\partial x^{2}}\\ +\left(-2C_{12}+2C_{22}-2C_{11}+2C_{21}\right)\frac{\partial w}{\partial x}+\left(-3C_{22}+4C_{31}-C_{11}\right)\frac{\partial^{3}w}{\partial x\partial \varphi^{2}}\\ +\left(C_{11}+C_{22}-2C_{31}\right)\frac{\partial^{4}w}{\partial x^{2}\partial \varphi^{2}}+\left(2C_{22}+2C_{21}-2C_{31}\right)\frac{\partial^{2}w}{\partial \varphi^{2}}+C_{21}\frac{\partial^{4}w}{\partial \varphi^{4}}+s_{1}^{}e^{x}\cot\gamma \left(\frac{\partial^{2}w}{\partial x^{2}}-\frac{\partial w}{\partial x}\right). \end{array}$$

## Appendix B

$$\begin{array}{l} a_{1}=-m_{2}^{2}A_{66}^{*}+A_{11}^{*}m_{1}^{4}+A_{22}^{*}m_{1}^{2}+2A_{12}^{*}m_{2}^{2}-5A_{11}^{*}m_{1}^{2}-2A_{22}^{*}m_{2}^{2}+A_{22}^{*}m_{2}^{4},\\ a_{2}=2m_{1}^{}m_{2}^{2}A_{66}^{*}-4A_{12}^{*}m_{1}^{}m_{2}^{2}+A_{22}^{*}m_{1}^{}-2A_{11}^{*}m_{1}^{}+4A_{11}^{*}m_{1}^{3},\\ a_{3}=A_{22}^{*}+A_{11}^{*}m_{1}^{2}+A_{66}^{*}m_{2}^{2}-2A_{12}^{*}m_{2}^{2}+A_{12}^{*}m_{2}^{2}+A_{22}^{*}m_{1}^{2}+A_{22}^{*}m_{2}^{4}-2A_{22}^{*}m_{2}^{2}+A_{11}^{*}m_{1}^{4},\\ a_{4}=2m_{1}m_{2}^{2}A_{66}^{*}-4A_{12}^{*}m_{1}m_{2}^{2},\\ a_{5}=\left(\begin{array}{l} C_{22}m_{1}^{2}m_{2}^{2}+C_{11}^{}m_{1}^{2}m_{2}^{2}-2C_{31}m_{1}^{2}m_{2}^{2}+C_{21}+C_{12}+C_{12}m_{1}^{4}+7C_{12}m_{1}^{2}+C_{21}m_{1}^{2}\\ +8C_{31}m_{2}^{2}-6C_{12}m_{1}^{2}-2C_{21}m_{2}^{2} \end{array}\right),\\ a_{6}=\left(\begin{array}{l} 8C_{31}m_{1}m_{2}^{2}-C_{11}m_{1}m_{2}^{2}-3C_{12}m_{1}+C_{22}m_{1}m_{2}^{2}+4C_{12}m_{1}^{3}+C_{11}m_{1}^{3}+C_{11}m_{1}\\ -4C_{12}m_{1}^{3}-C_{22}m_{1}^{3}-C_{22}m_{1}+4C_{12}m_{1} \end{array}\right). \end{array}$$

## Appendix C

$$M_{1}=\frac{1}{24}\frac{\pi^{2}m\left(\begin{array}{l} 96b_{6}x_{0}^{5}\sin\left(\gamma \right)+216b_{8}x_{0}^{5}\sin\left(\gamma \right)+12b_{1}e^{4x_{0}}\pi^{5}m^{5}S_{1}\cos\left(\gamma \right)\\ +16b_{3}e^{3x_{0}}\pi^{5}m^{5}S_{1}\cos\left(\gamma \right)+64b_{4}e^{3x_{0}}\pi mx_{0}^{4}\sin\left(\gamma \right)+80b_{4}e^{3x_{0}}\pi^{3}m^{3}x_{0}^{2}\sin\left(\gamma \right)\\ -150b_{8}e^{2x_{0}}\pi^{2}m^{2}x_{0}^{3}\sin\left(\gamma \right)-24b_{8}e^{2x_{0}}\pi^{4}m^{4}x_{0}^{}\sin\left(\gamma \right)+150b_{7}e^{2x_{0}}\pi^{3}m^{3}x_{0}^{2}\sin\left(\gamma \right)\\ +216b_{7}e^{2x_{0}}\pi mx_{0}^{4}\sin\left(\gamma \right)-24b_{6}e^{3x_{0}}\pi^{4}m^{4}x_{0}\sin\left(\gamma \right)-120b_{6}e^{3x_{0}}\pi^{2}m^{2}x_{0}^{3}\sin\left(\gamma \right)\\ +24b_{5}\pi^{4}m^{4}S_{1}x_{0}^{}\cos\left(\gamma \right)+120b_{5}\pi^{2}m^{2}S_{1}x_{0}^{3}\cos\left(\gamma \right)-39b_{1}\pi^{3}m^{3}S_{1}x_{0}^{2}\cos\left(\gamma \right)\\ -27b_{1}\pi mS_{1}x_{0}^{4}\cos\left(\gamma \right)+24b_{2}\pi^{4}m^{4}S_{1}x_{0}^{}\cos\left(\gamma \right)+78b_{2}\pi^{2}m^{2}S_{1}x_{0}^{3}\cos\left(\gamma \right)\\ -80b_{3}\pi^{3}m^{3}S_{1}x_{0}^{2}\cos\left(\gamma \right)-64b_{3}\pi mS_{1}x_{0}^{4}\cos\left(\gamma \right)+27b_{1}e^{4x_{0}}\pi mS_{1}x_{0}^{4}\cos\left(\gamma \right)\\ -24e^{4x_{0}}\cos(\gamma )\pi^{4}m^{4}S_{1}b_{2}x_{0}+39e^{4x_{0}}\cos(\gamma )\pi^{3}m^{3}S_{1}b_{2}x_{0}^{2}-78e^{4x_{0}}\cos(\gamma )\pi^{2}m^{2}S_{1}b_{2}x_{0}^{3}\\ -120e^{3x_{0}}\cos(\gamma )\pi^{2}m^{2}S_{1}b_{5}x_{0}^{3}+64e^{3x_{0}}\cos(\gamma )\pi mS_{1}b_{3}x_{0}^{4}-24e^{3x_{0}}\cos(\gamma )\pi^{4}m^{4}S_{1}b_{5}x_{0}\\ +80e^{3x_{0}}\cos(\gamma )\pi^{3}m^{3}S_{1}b_{3}x_{0}^{2}-54e^{4x_{0}}\cos(\gamma )S_{1}b_{2}x_{0}^{5}-96e^{3x_{0}}\cos(\gamma )S_{1}b_{5}x_{0}^{5}\\ +24e^{2x_{0}^{}}\pi^{5}m^{5}b_{7}\sin\left(\gamma \right)+16e^{3x_{0}^{}}\pi^{5}m^{5}b_{4}\sin\left(\gamma \right)-12\pi^{5}m^{5}b_{1}S_{1}\cos\left(\gamma \right)\\ -16\pi^{5}m^{5}b_{3}S_{1}\cos\left(\gamma \right)-216\pi mb_{7}x_{0}^{4}\sin\left(\gamma \right)+24\pi^{4}m^{4}b_{8}x_{0}^{}\sin\left(\gamma \right)\\ +120\pi^{2}m^{2}b_{6}x_{0}^{3}\sin\left(\gamma \right)-150\pi^{3}m^{3}b_{7}x_{0}^{2}\sin\left(\gamma \right)-80\pi^{3}m^{3}b_{4}x_{0}^{2}\sin\left(\gamma \right)\\ -64\pi mb_{4}x_{0}^{4}\sin\left(\gamma \right)+24\pi^{4}m^{4}b_{6}x_{0}^{}\sin\left(\gamma \right)+150\pi^{2}m^{2}b_{8}x_{0}^{3}\sin\left(\gamma \right)\\ -16\pi^{5}m^{5}b_{4}\sin\left(\gamma \right)-24\pi^{5}m^{5}b_{7}\sin\left(\gamma \right)-216e^{2x_{0}}b_{8}x_{0}^{5}\sin\left(\gamma \right)\\ -96e^{3x_{0}}b_{6}x_{0}^{5}\sin\left(\gamma \right)+96b_{5}S_{1}x_{0}^{5}\cos\left(\gamma \right)+54b_{2}S_{1}x_{0}^{5}\cos\left(\gamma \right) \end{array}\right)}{(4\pi^{2}m^{2}+9x_{0}^{2})\left(\pi^{2}m^{2}+x_{0}^{2}\right)\left(\pi^{2}m^{2}+4x_{0}^{2}\right)},$$

$$M_{2}=-\frac{1}{12}\frac{S_{1}^{4}m^{2}\pi^{3}\sin\left(\gamma \right)\left(e^{6x_{0}}-1\right)}{\pi^{2}m^{2}+9x_{0}^{2}},$$

$$M_{3}=-\frac{m^{2}\pi^{3}S_{1}^{2}\left(\begin{array}{l} \pi^{2}\sin\left(\gamma \right)e^{4x_{0}}-\pi^{2}\sin^{2}\left(\gamma \right)m^{2}+3e^{4x_{0}}\sin^{2}\left(\gamma \right)x_{0}^{2}+e^{4x_{0}}n^{2}x_{0}^{2}\\ -3\sin\left(\gamma \right)x_{0}^{2}-n^{2}x_{0}^{2} \end{array}\right)}{8\sin\left(\gamma \right)\left(\pi^{2}m^{2}+4x_{0}^{2}\right)x_{0}^{2}},$$

$$M_{4}=\frac{1}{4}\frac{\sin\left(\gamma \right)m\pi^{2}\left(e^{2x_{0}}\pi mb_{9}-e^{2x_{0}}b_{10}x_{0}-\pi mb_{9}+b_{10}x_{0}\right)}{x_{0}^{2}+\pi^{2}m^{2}},$$

$$M_{5}=-\frac{1}{12}\frac{S_{1}^{4}\pi m^{2}\sin\left(\gamma \right)\left(e^{6x_{0}}-1\right)}{m^{2}\pi^{2}+9x_{0}^{2}},$$

$$b_{1}=K_{1}m_{1}^{2}+3K_{2}m_{1}-2K_{1},b_{2}=K_{2}m_{1}^{2}-K_{1}m_{1}-K_{2},b_{3}=K_{3}m_{1}^{2}+K_{4}m_{1},$$

$$b_{4}=\left(\begin{array}{l} C_{11}K_{1}m_{1}^{2}m_{2}^{2}+C_{12}K_{1}m_{1}^{4}+C_{22}K_{1}m_{1}^{2}m_{2}^{2}-2C_{31}K_{1}m_{1}^{2}m_{2}^{2}+C_{11}K_{2}m_{1}^{3}+C_{11}K_{2}m_{1}^{}m_{1}^{2}\\ +4C_{12}K_{2}m_{1}^{3}-C_{22}K_{2}m_{1}^{3}+3C_{22}K_{2}m_{1}m_{2}^{2}-4C_{31}K_{2}m_{1}m_{2}^{2}-3C_{11}K_{1}m_{1}^{2}-5C_{12}K_{1}m_{1}^{2}\\ +C_{21}K_{1}m_{1}^{2}-2C_{21}K_{1}m_{2}^{2}+3C_{22}K_{1}m_{1}^{2}-2C_{22}K_{1}m_{2}^{2}+2C_{31}K_{1}m_{2}^{2}-2C_{11}K_{2}m_{1}\\ -2C_{12}K_{2}m_{1}+2C_{21}K_{2}m_{1}+2C_{22}K_{2}m_{1} \end{array}\right),$$

$$\begin{array}{l} b_{5}=K_{4}m_{1}^{2}-K_{3}m_{1},\\ b_{6}=\left(\begin{array}{l} C_{11}K_{2}m_{1}^{2}m_{2}^{2}+C_{12}K_{2}m_{1}^{4}+C_{21}K_{2}m_{2}^{4}+C_{22}K_{2}m_{1}^{2}m_{2}^{2}-C_{31}K_{2}m_{1}^{2}m_{2}^{2}-C_{11}K_{1}m_{1}^{3}\\ -C_{11}K_{1}m_{1}^{}m_{2}^{2}-4C_{12}K_{2}m_{1}^{3}+C_{22}K_{1}m_{1}^{3}-3C_{22}K_{1}m_{1}^{}m_{2}^{2}+4C_{31}K_{1}m_{1}^{}m_{2}^{2}-3C_{11}K_{2}m_{1}^{2}\\ -5C_{12}K_{2}m_{1}^{2}-2C_{12}K_{2}m_{2}^{2}+3C_{22}K_{2}m_{1}^{2}-2C_{22}K_{2}m_{2}^{2}+2C_{31}K_{2}m_{2}^{2}+2C_{11}K_{1}m_{1}^{}\\ +2C_{12}K_{1}m_{1}-2C_{21}K_{1}m_{1}-2C_{22}K_{1}m_{1} \end{array}\right), \end{array}$$

$$b_{7}=\left(\begin{array}{l} C_{11}K_{3}m_{1}^{2}m_{2}^{2}+K_{3}C_{12}m_{1}^{4}+K_{3}C_{21}m_{2}^{4}+K_{3}C_{22}m_{1}^{2}m_{2}^{2}-2K_{3}C_{31}m_{1}^{2}m_{2}^{2}+K_{4}C_{11}m_{1}m_{2}^{2}\\ -K_{4}C_{11}m_{1}m_{2}^{2}-K_{4}C_{22}m_{1}^{3}+K_{4}C_{22}m_{1}m_{2}^{2}+K_{3}C_{12}m_{1}^{2}+K_{3}C_{21}m_{1}^{2}-2K_{3}C_{21}m_{2}^{2}\\ +K_{4}C_{11}m_{1}-K_{4}C_{22}m_{1}+K_{3}C_{21} \end{array}\right),$$

$$b_{8}=\left(\begin{array}{l} C_{11}K_{4}m_{1}^{2}m_{2}^{2}+K_{4}C_{12}m_{1}^{4}+K_{4}C_{21}m_{2}^{4}+K_{4}C_{22}m_{1}^{2}m_{2}^{2}-2K_{4}C_{31}m_{1}^{2}m_{2}^{2}-K_{3}C_{11}m_{1}^{3}\\ +K_{3}C_{11}m_{1}m_{2}^{2}+K_{3}C_{22}m_{1}^{3}-K_{3}C_{22}m_{1}m_{2}^{2}+K_{4}C_{12}m_{1}^{2}+K_{4}C_{21}m_{1}^{2}-2K_{4}C_{21}m_{2}^{2}\\ -K_{3}C_{11}m_{1}+K_{3}C_{22}m_{1}+K_{4}C_{21} \end{array}\right),$$

$$b_{9}=-C_{24}m_{2}^{4}+\left(\left(2C_{32}-C_{14}-C_{23}\right)m_{1}^{2}+2C_{24}\right)m_{2}^{2}-C_{13}m_{1}^{4}+\left(-C_{13}-C_{24}\right)m_{1}^{2}-C_{24},$$

$$b_{10}^{}=\left(C_{23}-C_{14}\right)m_{1}m_{2}^{2}+\left(C_{14}-C_{23}\right)m_{1}^{3}+\left(C_{14}-C_{23}\right)m_{1}.$$
